# The PufX quinone channel enables the light‐harvesting 1 antenna to bind more carotenoids for light collection and photoprotection

**DOI:** 10.1002/1873-3468.12575

**Published:** 2017-02-10

**Authors:** John D. Olsen, Elizabeth C. Martin, C. Neil Hunter

**Affiliations:** ^1^ Department of Molecular Biology and BiotechnologyUniversity of SheffieldUK

**Keywords:** bacterial photosynthesis, carotenoid, light‐harvesting, membrane protein, quinones, reaction centre

## Abstract

Photosynthesis in some phototrophic bacteria requires the PufX component of the reaction centre–light‐harvesting 1–PufX (RC‐LH1‐PufX) complex, which creates a pore for quinone/quinol (Q/QH_2_) exchange across the LH1 barrier surrounding the RC. However, photosynthetic bacteria such as *Thermochromatium (T.) tepidum* do not require PufX because there are fewer carotenoid binding sites, which creates multiple pores in the LH1 ring for Q/QH_2_ exchange. We show that an αTrp_‐24_→Phe alteration of the *Rhodobacter (Rba.) sphaeroides* LH1 antenna impairs carotenoid binding and allows photosynthetic growth in the absence of PufX. We propose that acquisition of PufX and confining Q/QH_2_ traffic to a pore adjacent to the RC Q_B_ site is an evolutionary upgrade that allows increased LH1 carotenoid content for enhanced light absorption and photoprotection.

## Abbreviations

BChl(s), bacteriochlorophyll(s)

*Blc*,* Blastochloris*

Crt, carotenoid

cyt*bc*_*1*_, cytochrome *bc*_1_ complex

LH2, light‐harvesting LH2 complex

OD, optical density

Q/QH_2_, quinols/quinones

*Rba*., *Rhodobacter*

RC, reaction centre

RC‐LH1, reaction centre–light‐harvesting 1 complex

Rps., *Rhodopseudomonas*

*Rsp*., *Rhodospirillum*

*T*., *Thermochromatium*

WT, wild‐type

β‐DDM, β‐dodecylmaltoglucoside

The reaction centre–light‐harvesting 1 (RC‐LH1) complexes of purple phototrophic bacteria collect energy from sunlight, and in many bacteria, they also receive energy from LH2 antenna complexes, thereby increasing the light‐absorbing capacity of the photosystem. The RC traps this energy by initiating a series of electron transfers that eventually produce a quinol molecule, destined to leave the RC‐LH1 complex and diffuse to the cytochrome *bc*_1_ (cyt*bc*_1_) complex 1. Here, the original excitation energy is converted to a proton motive force. In some bacteria such as *Rhodospirillum (Rsp.) rubrum* and *Thermochromatium (T.) tepidum*, the LH1 antenna ring completely surrounds the reaction centre 2, 3, 4, but in others, such as *Rhodobacter (Rba.) capsulatus, Rba. sphaeroides* and *Rhodopseudomonas* (*Rps*.)* palustris*, the LH1 ring is interrupted by a PufX/PufW polypeptide 5, 6, 7. The structure of the dimeric *Rba. sphaeroides* RC‐LH1‐PufX complex 8 shows that PufX opens a small channel in the LH1 ring that allows quinols/quinones (Q/QH_2_) to cross the LH1 barrier and gain access to the RC Q_B_ site 8. This structure accounts for the abolished photosynthetic growth of PufX‐minus mutants 9, 10, 11, 12, 13, 14, as it appears that the space in the LH1 ring occupied by PufX is filled by two extra LH1 subunits that block access to the RC Q_B_ site 15. However, many bacteria do not have a PufX yet they still photosynthesise; the recent structure of the *T. tepidum* RC‐LH1 complex shows how this is possible, and inspection of the LH1 ring structure revealed a series of small pores that were suggested to allow Q/QH_2_ exchange in the absence of PufX 4; a similar situation could apply to *Rsp. rubrum*, as proposed earlier 16.

If, in principle, Q/QH_2_ can cross the LH1 barrier directly in *T. tepidum* and *Rsp. rubrum*, why do bacteria such as *Rba. sphaeroides* need to incorporate PufX into their LH1 rings to create a quinone channel? The explanation is proposed to originate in the carotenoid (Crt) contents of bacteria with and without PufX; for example, the *T. tepidum* and *Rsp. rubrum* LH1 complexes have a carotenoid: bacteriochlorophyll (BChl) ratio of 1 : 2 4, 17 and the LH1 complex is composed of α_1_β_1_BChl_2_Crt_1_ units, whereas this ratio is 1 : 1 for the *Rba. sphaeroides* LH1 18 and the LH1 complex is assembled from α_1_β_1_BChl_2_Crt_2_ units 8. We suggest that these ‘extra’ LH1 carotenoids confer significant advantages in terms of light harvesting and photoprotection and, although they would likely block the quinone pores identified in the ‘low‐carotenoid’ *T. tepidum* LH1 complex 4, PufX alleviates this potential problem by preventing LH1 from fully encircling the RC. Thus, the pore created by PufX allows Q/QH_2_ exchange 8, and the efficiency and robustness of the antenna are increased by increasing the carotenoid content.

In order to test this proposition, we constructed a genomic point mutation that lowers the carotenoid content of LH1, and combined it with genomic deletion of the *pufX* gene. The PufX^−^ strain DM1R, which harbours the LH1 αW_‐24_F mutation that lowers LH1 carotenoid content, grows photosynthetically, whereas the PufX^−^ mutant control with normal LH1 carotenoids cannot do so. We suggest that partial loss of carotenoid from LH1 α_1_β_1_BChl_2_Crt units encircling the RC in the DM1R mutant opens some pores in LH1 for quinone exchange, mimicking the ‘low‐carotenoid’ LH1 complexes of *Rsp. rubrum* and *T. tepidum* to a limited extent, and enabling this strain to tolerate the loss of PufX.

## Materials and methods

### *Rba. sphaeroides* strains

Genomic mutations were constructed in various combinations in the *Rba. sphaeroides* 2.4.1 WT using the suicide vector pk18mobsacB 19 as described 20, 21. These mutations were as follows: (a) deletion of the *pucBA1* and *pucBA2* genes encoding polypeptides of the LH2 complex, (b) deletion of the *pufX* gene and (c) site‐specific mutation of *puf*A to convert αTrp_‐24_→Phe (the numbering of LH1 residues relates to the conserved His_0_ residue that binds bacteriochlorophyll). Separately, we constructed a control strain DX13 harbouring a R_49_LR_53_L variant of PufX, which yields monomeric PufX‐containing RC‐LH1 complexes (Qian *et al*., in preparation; Table 1).

**Table 1:** *Rba. sphaeroides* strains used in this study

Strain	Description	Phenotype	Reference
DO10	Genomic deletion of *pucBA1, pucBA2, pufX*	LH2^−^, PufX^−^	This work
DM1R	Genomic deletion of *pucBA1, pucBA2, pufX*. Mutation in *pufA* giving W_‐24_F	LH2^−^, PufX^−^, LH1αW_‐24_F	This work
DX13	Genomic deletion of *pucBA1, pucBA2*. Mutation in *pufX* giving R_49_L, R_53_L.	LH2^−^, monomeric RC‐LH1‐PufX complexes	Qian *et al*., in preparation
RCO1	Strain DD13 (*pucBA1::Sm* ^*R*^ *; pufBALM::Kan* ^*R*^ *)* complemented *in trans* with pRKEH10D (*pufLMX*)	RC‐only	Jones *et al*. 1992 24

### Photosynthetic and semi‐aerobic growth of *Rba. sphaeroides* strains

The cells were grown in M22+ medium supplemented with vitamins and 0.1% casamino acids. The cells were grown either semi‐aerobically in the dark, as 1.5 L cultures in 2‐L conical flasks in a shaking incubator at 34 °C, or photosynthetically at room temperature in stirred 1‐L Roux bottles at ~ 50 μE light intensity.

The photosynthetic growth curves were recorded for cultures in 15‐mL flat‐bottomed screw cap tubes sealed with Parafilm and filled to capacity, and with a micro stir bar. The cultures were illuminated by halogen light bulbs to give a minimum light intensity of 50 μE. Growth was monitored with a WPA Colourwave CO7000 Medical Colorimeter equipped with a 680‐nm filter. Four separate cultures of each strain were grown simultaneously and their optical density values averaged for each time point. Each inoculum, using a semi‐aerobically grown culture of each strain, was adjusted so that each culture started with the same number of cells.

### Pigment extraction

Cells from 1 mL of an 80 mL semi‐aerobic culture of the relevant strains were pelleted in a microcentrifuge and extracted twice into 0.5 mL of acetone : methanol (7 : 2 v/v). Absorption spectra of pooled 1 mL samples of extracts were recorded using a Cary 60 spectrometer. The peak heights at 478 nm (carotenoids) and 770 nm (BChls) were measured; three replicates of three separate semi‐aerobic cultures were measured to obtain the final averaged data. All procedures were conducted under dim light conditions.

### Membrane preparation and low‐temperature spectroscopy

Membranes were prepared as described in Olsen *et al*. 22 and kept at −20 °C prior to use. Low‐temperature (80K) absorbance spectra were measured using a DN10 Cryostat (Oxford Instruments, Abingdon, UK) mounted in a Cary 60 spectrophotometer (Agilent, Stockport, UK).

### Fractionation of photosynthetic complexes on sucrose density gradients

Membranes were fractionated as described in 21. Samples were solubilised in 3% β‐DDM, fractionated on a discontinuous sucrose gradient containing 20, 21.25, 22.5, 23.75 and 25% sucrose in 20 mm HEPES and 0.03% β‐DDM, then centrifuged in a Beckman SW41 Ti rotor at 27 000 rpm (90 000 ***g***) for 40 h.

## Results

### Photosynthetic growth rates of DM1R, and PufX^+^ and PufX^−^ control strains

In order to lower the carotenoid content of the *Rba. sphaeroides* LH1 complex, thereby producing at least a few of the pores observed in the low‐carotenoid *T. tepidum* RC‐LH1 complex 4, the LH2^−^, PufX^−^ strain DM1R was constructed in which αTrp_‐24_ at the N terminus of the LH1α polypeptide was changed to Phe (LH1αW_‐24_F). This mutation is similar to the αTrp_‐24_ to Tyr alteration in *Rba. capsulatus* that lowered the carotenoid content of LH1 by 60% 23. In order to simplify spectral analyses, all strains harboured genomic deletions of *pucBA1, pucBA2* that remove LH2 complexes.

**Figure 1:**
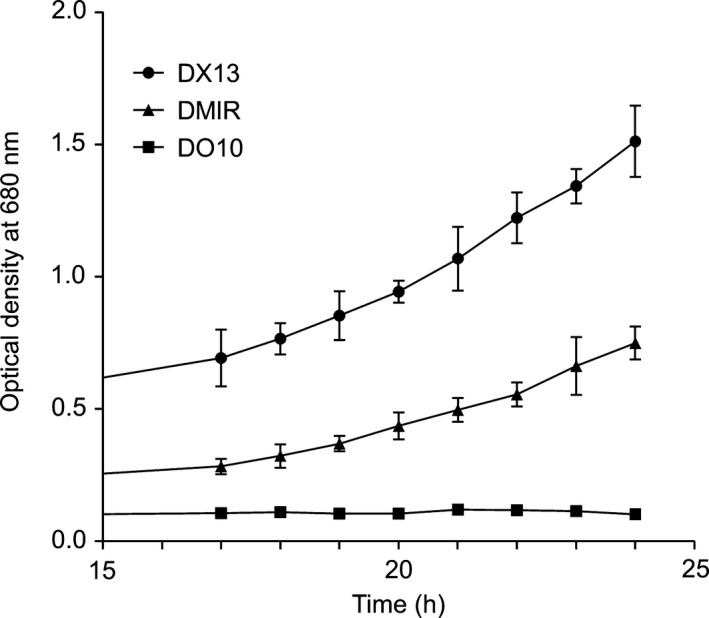
Photosynthetic growth curves of DM1R, DO10 and DX13 strains, with four biological replicates for each time point. DX13 is the positive control PufX^+^ strain; DM1R is PufX^−^, LH1αW_‐24_F; DO10 is a PufX^−^ negative control.

DM1R was tested for photosynthetic growth against the two LH2^−^ control strains: DO10, which is the same as DM1R, that is PufX^−^ but with normal LH1 complexes, and DX13, which is PufX^+^ but with monomeric RC‐LH1‐PufX core complexes to match the monomeric RC‐LH1 core complexes in DM1R and DO10. Absorption of whole cells (not shown) showed that DM1R has 61% of the LH1 content of DO10, which likely arises from some impairment of LH1 assembly by the LH1αW_‐24_F alteration. DM1R grew more slowly than the positive control PufX^+^ strain DX13, likely because DX13 has an optimised quinone channel created by PufX, but significantly faster than the PufX^−^ LH2^−^ control strain DO10, which was photosynthetically inactive as expected (Fig. 1A). Equally inactive is the RC‐only (LH2‐minus, LH1‐minus) strain RCO1 24, which shows no photosynthetic growth within the same 24‐h period used in Fig. 1, and at the same 50 μE light intensity (results not shown).

### Biochemical and spectroscopic analyses of the DM1R and DO10 strains

**Figure 2:**
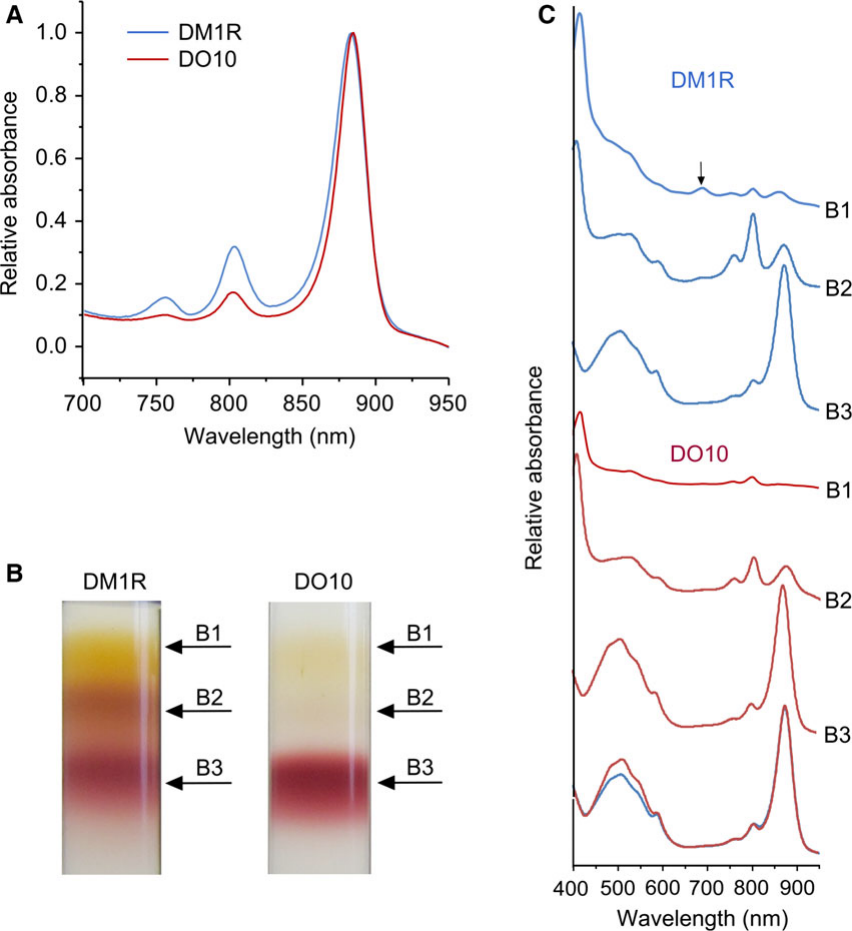
(A) Low‐temperature absorbance spectra of DM1R and DO10 membrane samples recorded at 77K, normalised at the LH1 absorption maximum near 875 nm. (B) Fractionation of detergent‐solubilised intracytoplasmic membranes from DM1R and DO10 strains on sucrose density gradients. (C) Room temperature absorbance spectra of the B1, B2 and B3 fractions from the gradients in panel B. The arrow in the DM1R B1 fraction indicates absorption at ~ 680 nm; overlaid spectra (bottom) compare the two B3 fractions.

The ability of DM1R to grow photosynthetically could be explained if the LH1αW_‐24_F alteration destabilises the formation of a complete LH1 ring; then, missing LH1 α_1_β_1_ units could create gaps for Q/QH_2_ exchange. In order to investigate this point, we performed biochemical and spectroscopic analyses of the PufX^−^ DM1R and DO10 strains. The low‐temperature (77K) absorption spectra in Fig. 2A were normalised for LH1 absorption, to emphasise the equivalence of the core complexes in terms of LH1 : RC stoichiometry (see below), and to reflect the presence of some RCs with no LH1, which appear in fractions B1 and B2 (Fig. 2B,C).

To analyse differences between DM1R and DO10 strains in more detail, comparable amounts of DM1R and the DO10 control membranes, in terms of RC content, were solubilised with β‐dodecylmaltoglucoside (β‐DDM) and then fractionated by centrifugation through sucrose density step gradients. Figure 2B shows that β‐DDM releases some ‘free’ RCs from DM1R membranes, as shown by the distinctive absorption spectrum of these RC complexes (Fig. 2C,B1,B2, blue). The equivalent fractions from the DO10 control, barely visible in the images of the gradients (Fig. 2B), have a similar absorption spectrum (Fig. 2C,B1,B2, red). These spectra show that the limited impairment of LH1 assembly by the LH1αW_‐24_F alteration in DM1R has left some RCs with no encircling LH1 antenna. Another likely consequence of lessened LH1 assembly is seen in the B1 fractions; some RCs are present in each case, but relative to DO10 the B1 fraction in DM1R has more unattached carotenoid and also a small absorption peak at ~ 680 nm (Fig. 2C, arrowed), possibly from an intermediate of BChl biosynthesis.

If the LH1 ring was interrupted in DM1R, due to incomplete assembly of LH1 α_1_β_1_ subunits round the RC, there would be space for Q/QH_2_ exchange despite the loss of PufX and this would explain the photosynthetic growth of DM1R in Fig. 1A. However, the cores that do assemble in DM1R have a normal LH1 : RC stoichiometry, as shown by the close correspondence between the overlaid spectra of the B3 fractions for DM1R and the DO10 positive control (Fig. 2C, bottom). These two spectra have almost identical 874 : 805 nm (LH1 : RC) absorption ratios so these two PufX^−^ mutants, DM1R and DO10, have a complete ring of LH1 subunits encircling the RC. The only difference between the RC‐LH1 complexes of DM1R and DO10 is seen in the 450‐ to 550‐nm carotenoid region where there is clear evidence for lowered carotenoid content, 14% lower when measured at 508 nm, as a consequence of the W_‐24_F alteration of LH1 in DM1R.

In summary, the lowered carotenoid content of the LH1 antenna (Fig. 2C, bottom spectra), a consequence of the LH1αW_‐24_F mutation, is the likely explanation for the acquisition of photosynthetic growth in the absence of PufX. The presence of LH1‐free RCs cannot account for the photosynthetic growth of the PufX^−^ strain DM1R because the antenna‐free, RC‐only strain RCO1 is photosynthetically inactive under the same light conditions (results not shown).

### The DM1R mutant has a lowered carotenoid content relative to LH1 BChl pigments

The appearance of more carotenoid in the B1 fraction in the DM1R gradient (Fig. 2B,C) shows that some of this pigment, destined for binding to LH1, is unattached to any complex. The presence of some ‘free’ carotenoid in the B1 fraction of the DO10 control shows that carotenoid biosynthesis is not perfectly coupled to complex assembly. In order to verify the loss of some carotenoid from the LH1 of the DM1R mutant (Fig. 2C), DM1R (LH1αW_‐24_F, PufX^−^) and the DO10 (WT LH1, PufX^−^) control were grown semi‐aerobically and the carotenoid and bacteriochlorophyll (BChl) pigments were extracted from whole cells with acetone/methanol. The 478 : 770 nm absorption ratios for three biological replicates of each strain were 1.38 ± 0.12 and 1.80 ± 0.08, respectively, so the carotenoid content of the DM1R strain is 23% lower with respect to DO10, on the basis of equal BChl content. The lower LH1 content per cell in DM1R, which is 61% of that in DO10, is taken into account by comparing 478 : 770 nm absorption ratios for pigments extracted from these two strains. Full occupancy of pigment binding sites in the RC‐LH1 complex of DO10 equates to 33 Crt (32 LH1 Crt and 1 RC Crt) and 36 BChl (32 LH1 BChl and 4 RC BChl). The appreciable population of LH1‐free RCs in DM1R, each with four BChl and one Crt, skews the overall BChl : Crt ratio towards a higher number. Conversely, the presence of more ‘free’ carotenoids in DM1R (Fig. 2B,C), which are included in the extractions of whole cells, leads to an overestimation of LH1‐bound carotenoids in DM1R. Overall, we conclude that the W_‐24_F alteration of LH1 in DM1R has impaired the binding of carotenoids, as also found in the earlier study 23 on a mutant of *Rba. capsulatus* harbouring an LH1αW_‐24_Y alteration.

## Discussion

It has long been known that PufX^−^ mutants of *Rba. sphaeroides* are photosynthetically inactive 9, 11, 12, 13, 14, 25 because they assemble an LH1 ring that completely encloses the RC 25, 26. This observation has been difficult to reconcile with bacteria with similarly closed LH1 antenna rings such as *Rsp. rubrum, T. tepidum* and *B. viridis* 2, 4, 27, 28, which do grow photosynthetically. Aird *et al*. 16 conducted a steered molecular dynamics simulation study of the LH1 complex of *R. rubrum* and proposed that the LH1 ring could allow quinones to move across the complex. Subsequently, the 3D structure of *T. tepidum* 4 provided a structural basis for this proposal by identifying pores in the LH1 ring for Q/QH_2_ exchange. In the present work, we set out to engineer a few such pores in the LH1 ring of *Rba. sphaeroides*, having first deleted the *pufX* gene in order to close the normal pore created by the PufX polypeptide 8. We were guided by the earlier mutagenesis work of Babst *et al*. 23, who showed that the LH1αW_‐24_Y mutation results in the loss of 40% of the carotenoids from the LH1 complex of *Rba. capsulatus*. Here, we show that alteration of LH1αW_‐24_ to F in the DM1R mutant, which lowers the cellular carotenoid content and also the level of carotenoids bound by the DM1R LH1 complex, restores some photosynthetic growth to a PufX^−^ LH2^−^ strain of *Rba. sphaeroides*.

**Figure 3:**
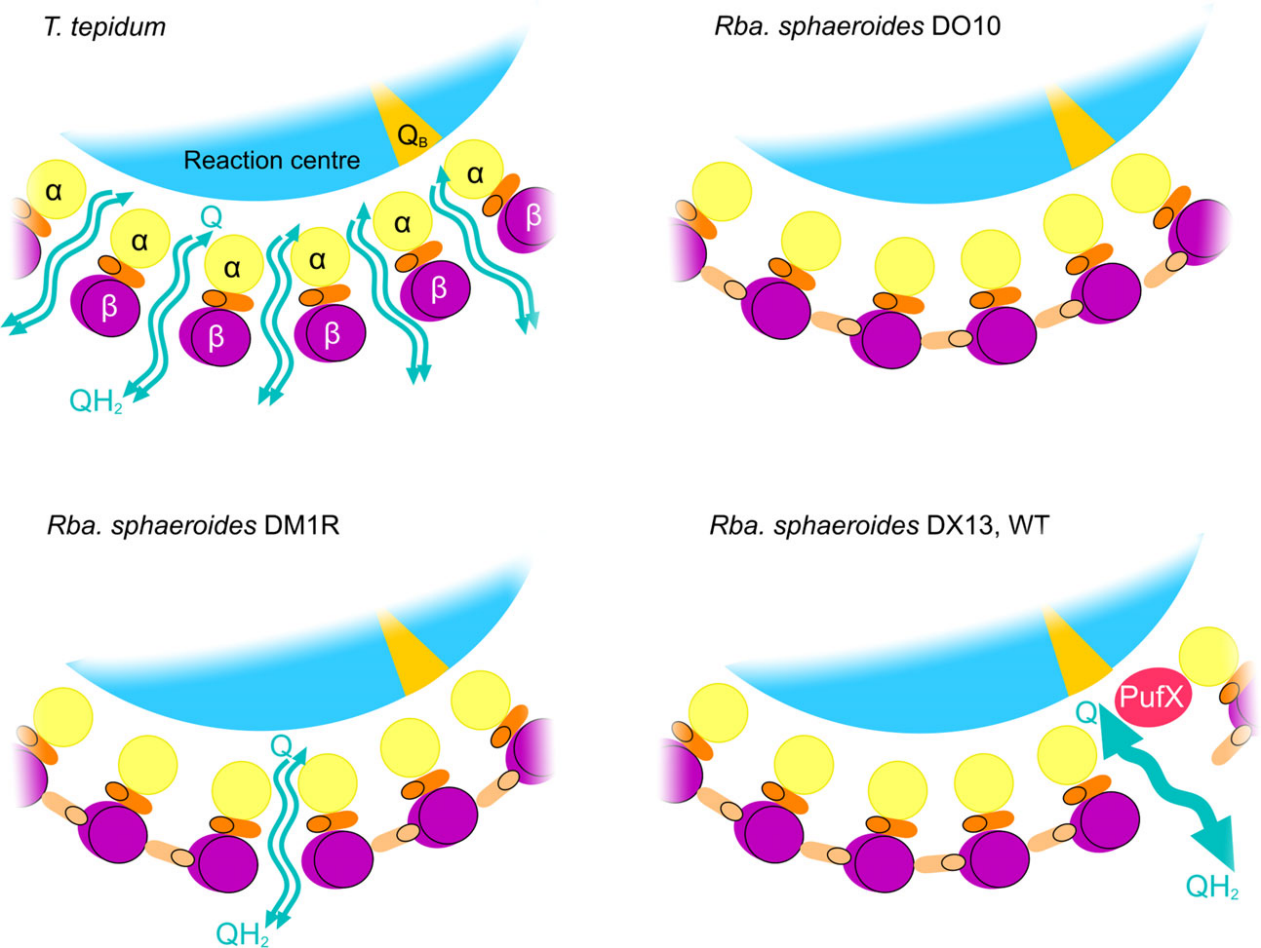
Schematic representation of part of the RC‐LH1 complex of *T. tepidum*,* Rba. sphaeroides* control strains DO10 and DM13, as well as the lowered LH1 carotenoid strain DM1R. The schematic depicts a section through the LH1 ring at the level of putative quinone pores between adjacent LH1 α_1_β_1_ BChl_2_ units. Carotenoids are depicted as foreshortened cylinders, viewed end‐on. For simplicity, BChls are not shown. In *T. tepidum*, only one of the two carotenoid binding sites is occupied for each α_1_β_1_ BChl_2_ LH1 unit; we propose that in *Rba. sphaeroides* the carotenoid (shown in dark orange) between the α and β LH1 apoproteins corresponds to the binding site seen in the *T. tepidum* structure. In *Rba. sphaeroides* strain DO10 (WT LH1, PufX^−^) full occupancy of carotenoid binding sites in LH1 prevents formation of *T. tepidum*‐like pores. Lowering the LH1 carotenoid content in strain DM1R impairs full occupancy of the carotenoid binding sites and passage of quinones through one or more pores is sufficient to permit some photosynthetic growth; 86% of the LH1 carotenoids are retained in DM1R with respect to DO10, so the assumption is made that most carotenoid binding sites are not affected by the alteration of LH1αW_‐24_ to F in the DM1R mutant. In the DX13 and WT strains, the PufX polypeptide creates a quinone pore, which allows the assembly of 14 α_1_β_1_ BChl_2_Crt_2_ units round the RC.

The structural basis for lowered carotenoid levels in the LH1αW_‐24_F complex has not been established, as carotenoid binding sites are poorly defined in the *Rba. sphaeroides* RC‐LH1‐PufX complex. This mutation also affects the assembly of the LH1 complexes to a limited degree, which results in some ‘free’ RCs in the membrane with no surrounding LH1 ring; similar effects were observed in previous studies of LH1 assembly mutants of *Rba. sphaeroides* 21, 29. The PufX‐minus RC‐LH1 complexes in the DM1R mutant assemble closed LH1 α_16_β_16_ rings because co‐operative forces between the RC and LH1 α_1_β_1_BChl_2_ subunits drive the process of encircling RCs with LH1 α_1_β_1_BChl_2_Crt_2_ units to completion, at the expense of some ‘free’ RCs that have no LH1 subunits. This LH1 ring completion process is observed even under quite extreme circumstances; for example, only ~ 14% of the LH1α polypeptide survives deletion of the LH1 assembly factor LhaA yet complexes still assemble with a normal RC : LH1 stoichiometry, leaving a substantial population of ‘free’ RCs 21. In summary, we propose that the LH1 ring of the PufX^−^ LH1αW_‐24_F mutant DM1R completely surrounds the RC, on the basis of the near‐identical RC : LH1 absorption ratios for DM1R and the control strain DO10 (Fig. 2C, bottom). The LH1‐free RCs in DM1R require no PufX to facilitate Q/QH_2_ turnover at the RC Q_B_ site, but they are unable to function at the low light intensities used because they have no antenna to collect and deliver excitation energy; this is why the control RC‐only strain RCO1 shows negligible photosynthetic growth. Thus, the observed growth rate of DM1R likely arises from the lowered carotenoid content of the RC‐LH1 complexes and the ensuing pores for Q/QH_2_ traffic, which are depicted in Fig. 3.

Phototrophic bacteria such as *T. tepidum* and *Rsp. rubrum* possess monomeric LH1 complexes that completely encircle the RC but small pores in each LH1 complex, created by having only a single carotenoid for each α_1_β_1_BChl_2_ subunit, likely allow Q/QH_2_ exchange and therefore turnover at the RC Q_B_ site 4, 16. Figure 3 shows schematic diagrams of a section of the LH1 ring of the control strain DO10, the reduced carotenoid mutant DM1R, *T. tepidum* and the core monomer mutant DX13, which in this context is equivalent to the WT. We illustrate the proposed gaps between LH1 α_1_β_1_BChl_2_Crt units that allow Q/QH_2_ exchange across the *T. tepidum* LH1 ring 4, and suggest that in *Rba. sphaeroides* the second carotenoid within each LH1 α_1_β_1_BChl_2_ unit effectively plugs these pores and prevents Q/QH_2_ exchange. This problem in the PufX^−^ strain DO10 is partly alleviated by the ~ 14% reduction in the carotenoid content of LH1 in strain DM1R, which creates a few pores for passage of Q/QH_2_. The optimal configuration is found in DX13 and the WT, where Q/QH_2_ traffic is confined to a site adjacent to the RC Q_B_ site, and a doubling of carotenoid content provides enhanced light absorption by the LH1 ring.

In relation to monomeric cores lacking PufX, the dimeric RC‐LH1‐PufX complex of *Rba. sphaeroides* has several functional upgrades that improve its light‐harvesting and quinone exchange functions. First, PufX binds to the RC‐H subunit and creates a single, specialised channel for efficient quinone traffic across the LH1 ring at a strategic location, relatively close by the RC Q_B_ site 8. Second, the LH1 complex is freed from maintaining small pores for quinone exchange across LH1 α_1_β_1_BChl_2_Crt units, so allowing recruitment of more carotenoids that enhance the light‐harvesting and photoprotective functions of the LH1 complex. Photoprotective effects are initiated by the oxygen‐driven *in situ* modification of spheroidene to yield spheroidenone 30, 31, 32, activating an intermolecular charge‐transfer state that channels excitation energy to the LH1 BChls whilst optimising the triplet energy for singlet oxygen quenching 33. This highly effective photoprotective mechanism occurs even in LH1 complexes that have been removed from the native membrane 34. Third, PufX‐promoted dimerisation of the RC‐LH1‐PufX complex confers additional benefits by allowing excitation and quinone sharing, as originally proposed by Comayras *et al*. 35 and shown directly in the structure of the dimeric RC‐LH1‐PufX complex 8.
